# The role of 18F-fluorodeoxyglucose positron emission tomography/computed tomography SUV_Max_ in deciding on a computed tomography-guided lung biopsy in solid solitary pulmonary nodules

**DOI:** 10.1590/1806-9282.20241741

**Published:** 2025-05-02

**Authors:** Bekir Sıtkı Said Ulusoy, Ali Burak Binboga, Mehmet Onay, Cetin Murat Altay, Ahmet Burak Kara

**Affiliations:** 1 University of Health Sciences, Gaziantep City Hospital, Department of Radiology – Gaziantep, Turkey.

**Keywords:** Solitary pulmonary nodule, Biopsy, Needle, CT scans, Fluorodeoxyglucose F18

## Abstract

**OBJECTIVE:**

The aim of this study was to calculate a useful cut-off point of the ^18^F-fluorodeoxyglucose positron emission tomography/computed tomography SUV_Max_ value to decide on a computed tomography-guided percutaneous transthoracic needle biopsy for solitary pulmonary nodules of sizes between 11 and 20 mm.

**METHODS:**

Between January 2015 and April 2020, patients with solitary pulmonary nodules who underwent computed tomography-guided percutaneous transthoracic needle biopsy were retrospectively reviewed, and those with solitary pulmonary nodules of 11–20 mm in diameter, who had undergone an ^18^F-fluorodeoxyglucose positron emission tomography/computed tomography examination before computed tomography-guided percutaneous transthoracic needle biopsy, were included in the study. A total of 76 patients who met the inclusion criteria were evaluated.

**RESULTS:**

There was no distinguishing finding on the computed tomography examination (p>0.05). The SUV_Max_ values of the malignant solid solitary pulmonary nodules were higher than the benign solitary pulmonary nodules (p<0.05).

**CONCLUSION:**

The benign and malignant solid solitary pulmonary nodules between 11 and 20 mm have similar computed tomography features. ^18^F-fluorodeoxyglucose positron emission tomography/computed tomography is a useful imaging technique for distinguishing benign and malignant solitary pulmonary nodules. Notably, 4.85 SUV_Max_ value can be used to decide on a computed tomography-guided percutaneous transthoracic needle biopsy procedure in solid solitary pulmonary nodules between 11 and 20 mm with excellent sensitivity and moderate specificity rates.

## INTRODUCTION

A solitary pulmonary nodule (SPN) is a single, spherical, or oval-shaped radiopaque lesion smaller than 30 mm in diameter, which is completely surrounded by pulmonary parenchyma^1^. It was reported that changes in the diagnostic yield of computed tomography biopsy significantly altered preferences when the probability of malignancy was 10 or 30% of current smokers or ex-smokers^2^. The malignancy rate is approximately 30% for SPNs with a diameter of 21–30 mm and approximately 12% for those with a diameter of 11–20 mm^3^. The 5-year survival rate for lung cancer is approximately 15.6% since it is diagnosed in an advanced stage in most patients^4^. Therefore, the early diagnosis of malignant SPNs is critical to increase the survival of lung cancer patients.

CT-PTNB is a safe and effective minimally invasive procedure with high diagnostic accuracy^5^. Nodule size is one of the major risk factors for increased complication rates^6^. The smaller size of SPNs increases the failure rate of accessing the target lesion, the rate of insufficient tissue sampling, and the rate of false-negative results, and it is also linked to a higher complication rate^7,8^. Therefore, it may be difficult to decide on a CT-PTNB procedure due to the higher risk of complications and inadequate biopsy results, especially in SPNs under 20 mm. As an alternative to tissue sampling in managing solid SPNs larger than 8 mm, regardless of risk group, a 3-month thorax CT and/or PET/CT follow-up is recommended by the Fleischner Society guidelines^9^.

^18^F-FDG PET/CT is a metabolic imaging method routinely performed in oncology practice^10^. ^18^F-FDG PET/CT has been successfully used to investigate SPNs since 2000s^11^. In literature, although many publications have reported the role of ^18^F-FDG PET/CT, there is no consensus on the cut-off value of SUV_Max_ in different size pulmonary nodules.

The aim of this study was to calculate a useful cut-off point of the ^18^F-FDG PET/CT SUV_Max_ value to decide on a CT-PTNB for solid SPNs of sizes between 11 and 20 mm.

## METHODS

This study conformed in accordance with the 2013 Declaration of Helsinki. The study was approved by the Ethics Committee of Gaziantep University (No: 2020/326). Written informed consent was obtained from all individual participants included in the study.

### Patients

All biopsy procedures were decided considering the Fleischner Society 2013 and 2017 criteria^9^. Between January 2015 and April 2020, patients with SPNs who underwent CT-PTNB in the interventional radiology department of our hospital were retrospectively reviewed. A total of 112 patients with SPNs who had complete CT-PTNB reports were found in our interventional radiology database. In total, 36 of these patients were excluded from the study due to having SPNs larger than 20 mm in size (n=17), having subsolid nodules (n=13), or not undergoing an ^18^F-FDG PET/CT examination despite having SNPs of 11–20 mm in diameter (n=6). Finally, a total of 76 patients (53 males and 23 females, mean age 62.21±12.69 years), who met the inclusion criteria were evaluated.

### Biopsy procedure

Biopsies were performed on patients with INR <1.5 and platelet count >50,000 K/μL. Positioning (supine, prone, or lateral) was adjusted based on SPN location for safe needle access. Using a Siemens CT (SOMATOM) with 20 mAs, 120 kV, 8×1.25 mm collimation, and 2.5 mm slice thickness, each procedure employed an 18-G cutting needle and a 17-G coaxial needle. At least three cuts were taken for adequate tissue sampling, and a post-procedural CT was conducted to check for complications such as pneumothorax or hemorrhage.

### Diagnostic evaluation

CT-PTNB diagnoses were categorized as malignant, benign, unspecified (atypical cells), and non-diagnostic (insufficient tissue). Final diagnoses were confirmed via surgical resection, follow-up CT, or lab/pathology exams. A ≥20% reduction in nodule size on follow-up CT signified a benign SPN.

### Computed tomography images

Diagnostic contrast-enhanced or non-contrast thorax CT images were reviewed by two radiologists, each with 5 years of experience, who were blinded to the pathologic diagnosis. The CT morphological features of the solid SPNs, nodule diameter (mm), nodule contour (smooth, lobulated, spiculated), localization, hilar lymphadenopathy (LAP), and emphysema were determined based on consensus.

### ^18^F-fluorodeoxyglucose positron emission tomography/computed tomography images

All ^18^F-FDG PET/CT images were routinely obtained after at least 6 h of fasting. The ^18^F-FDG PET/CT images from the skull base to the middle of the thigh were obtained approximately 1 h later in patients who received ^18^F-FDG once their serum glucose levels were <110 mg/dL. All collected data were transferred to dedicated workstations for post-processing, and the SUV_Max_ values of the SPNs were calculated and noted separately.

### Statistical analysis

Data were analyzed using SPSS 21.0 (IBM Corp., Armonk, NY, USA). Frequency distributions were provided for categorical variables, and means, standard deviations, medians, and IQRs for numerical variables. The Mann-Whitney U test, t-test, and chi-square test assessed differences between groups, while ROC analysis determined the SUV_Max_ cut-off value. A p-value <0.05 indicated statistical significance.

## RESULTS

### Biopsy-based diagnosis and final diagnosis

Sufficient tissue was obtained for pathological examination in all patients except one with a 12 mm solid SPN in the right lower lobe. The coaxial CT-PTNB diagnoses were malignant (81.5%), benign (14.5%), unspecified (2.7%), and non-diagnostic (1.3%). Final diagnoses were malignant (81.5%), benign (17.2%), and missing (1.3%). Malignant diagnoses were confirmed by surgical resection, while benign findings were accepted as final (Table 1).

**Table 1:** Coaxial computed tomography-guided percutaneous transthoracic needle biopsy and final diagnoses of the 76 solid solitary pulmonary nodules of 11–20 mm in diameter.

Diagnoses	CT-PTNB diagnoses		Final diagnoses	
	n	%	n	%
Adenocarcinoma	31	40.8	31	40.8
Squamous cell carcinoma	14	18.4	14	18.4
Small cell carcinoma	7	9.2	7	9.2
Metastasis	10	13.2	10	13.2
Granulomatous inflammation	5	6.5	5	6.5
Benign cytology	5	6.5	7	9.2
Hydatid cyst remnant	1	1.3	1	1.3
Unspecified atypical cells*	2	2.7	None	None
Non-diagnostic?	1	1.3	Missing	Missing

* Final diagnoses were benign;

? missing.

CT-PTNB: computed tomography-guided percutaneous transthoracic needle biopsy.

There was a statistically significant relationship between pathological diagnosis and gender. The malignancy rate was higher in males than in females (p=0.037). A statistically significant difference was also observed between the pathological diagnosis and the ^18^F-FDG PET/CT SUV_Max_ median values. The median SUV_Max_ value of the malignant SPNs was significantly higher than that of the benign SPNs (med-IQR 11.2–7 vs. 6.9–13.5, p=0.006). The patient age and the CT features of the SPNs (size, contour, and presence of hilar LAP and emphysema) were similar in benign and malign cases (p>0.05) (Table 2).

**Table 2:** Comparison of the computed tomography and ^18^F-fluorodeoxyglucose positron emission tomography/computed tomography findings of the benign and malignant solid solitary pulmonary nodules.

	Benign vs. malignant SPN	
	Test value	p-value
Sex	4.748^c^	0.037**
Age	1.308^mw^	0.191
Nodule size	1.043^mw^	0.297
Contour	0.332^c^	0.564
Hilar LAP	1.359^c^	0.244
Emphysema	0.646^c^	0.421
^18^F-FDG PET/CT SUV_Max_	2.734^mw^	0.006**

mw Mann-Whitney U test;

c Chi-square test;

** statistically significant.

SPN: solitary pulmonary nodule; LAP: lymphadenopathy; ^18^F-FDG: ^18^F-fluorodeoxyglucose; PET/CT: positron emission tomography/computed tomography.

The ROC curve analysis was performed to obtain a cut-off value for ^18^F-FDG PET/CT SUV_Max_ indicating the malignancy risk of solid SPNs. According to the ROC analysis, two cut-off values were identified for SUV_Max_ indicating malignancy risk: 7.05 and 4.85. At the cut-off value of 7.05, SUV_Max_ had a sensitivity of 93.1% and specificity of 83.3%. At the cut-off value of 4.85, the sensitivity and specificity of SUV_Max_ were calculated as 100 and 66.7%, respectively.

## DISCUSSION

Although the diagnostic accuracy of CT-PTNB is very high in large nodules, in SPNs smaller than 20 mm, the diagnostic accuracy rates decrease, and reaching the target lesion with a needle can be challenging^8,12^. Another disadvantage of CT-guided biopsy in SPNs smaller than 20 mm is increased complication rates^13^. The likelihood of malignancy increases in large nodules, but nodule size does not exclude malignancy^9^. However, lipoid pneumonia, focal atelectasis, granulomatous inflammation, and progressive massive fibrosis, which are benign conditions, can also exhibit spiculated contours^14^. Although well-defined margins and a smooth contour may be a sign of benignity, pulmonary metastasis and 20% of primary lung malignancies have smooth contours^15^. Therefore, it becomes more challenging to decide on a biopsy procedure for SPNs smaller than 20 mm.

The role of ^18^F-FDG PET/CT is clearer in SPNs with a 20–30 mm size compared to SPNs of 11–20 mm in diameter due to the very low specificity, low spatial resolution, and partial volume effect^16^. Malignant nodules have higher ^18^F-FDG uptake values than benign nodules^17,18^. In other studies, the sensitivity and specificity of SUV_Max_ ≥2.5 were reported as 77 and 85%, respectively, for>8 mm nodules, and as 95 and 46%, respectively, for those smaller than 30 mm^19,20^. While the efficiency of ^18^F-FDG PET/CT is rather limited in lung nodules with pure ground-glass opacity, the reliability of ^18^F-FDG PET/CT increases in nodules with a solid component larger than 5 mm^21^. Another factor may be related to the necrosis content of the nodule. Due to the smaller necrosis area in small nodules, the ^18^F-FDG uptake increases, and PET/CT gives a higher SUV_Max_ value^16^. Therefore, a wide necrotic component of larger nodules may mislead the physicians based on low SUV_Max_ values. Furthermore, this heterogeneity of lung nodules may make it difficult to determine a cut-off value for SUV_Max_ to distinguish benign and malignant nodules at high sensitivity and specificity rates.

Chronic granulomatous inflammation may exhibit spiculated contours on CT similar to malignant SPNs, and this decreases the specificity of ^18^F-FDG PET/CT^20,22^. The sensitivity and specificity rates of ^18^F-FDG PET/CT decrease in pure GGNs and subsolid nodules that have a solid component smaller than 10 mm due to the low ^18^F-FDG uptake^23^. Dabrowska et al.^18^ investigated a cut-off value of ^18^F-FDG PET/CT to identify malignant SPNs in 71 patients. They determined that at a cut-off value of 2.1, SUV_Max_ had 77% sensitivity and 92% specificity^18^. The SUV_Max_ value may decrease to 1.25 in subsolid nodules, and the low SUV_Max_ value of benign nodules creates an overlap zone for the SUV_Max_ value in distinguishing benign and malignant SPNs^16^. This overlap zone reduces the specificity rates of ^18^F-FDG PET/CT.

The rates of major and overall complications of CT-PTNB are reported as 5.7% and 38.8%, respectively^24^. In addition, emphysema and needle path length are mentioned as the risk factors of pneumothorax^13,25^.

This study has certain limitations that should be noted. First, the small sample size of the study makes it difficult to interpret the data. Furthermore, due to the retrospective nature and single-center design of the study, the data may be subject to bias. Multicenter, prospective, randomized studies with larger samples are needed.

## CONCLUSION

Solid pulmonary nodules of 11–20 mm often have similar CT features, complicating benign versus malignant differentiation. The ^18^F-FDG PET/CT, with an SUV_Max_ threshold of 4.85, improves diagnostic accuracy and helps guide CT-guided biopsies. This threshold ensures high sensitivity and balanced specificity, supporting precise biopsy decisions and enhancing patient-centered care for small SPNs.
